# 

**Published:** 1910-03

**Authors:** 


					CONTENTS. pagt
Radium and some of its Physical and Therapeutic Properties.
By James Mackenzie Davidson, M.B., C.M.
Diffuse Destructive Changes in the Liver Associated with Jaundice.
By Carey Coombs, M.D., M.R.C.P. Lond.
17
Infection of the Urinary Tract with Bacillus Coll.
By J. R. Charles, M.A., M.D. Cantab., M.R.C.P.
23
Hypn otism.
By Hdgh P. Costobadie, M.R.C.S., L.R.C.P.
33
Oriental Medicine?Stray Jottings.
By V. B. Green-Armytage, I.M.S.
4?
Progress of the Medical Sciences:
Medicine.
Carey Coombs, M.D., M.R.C.P. Lond.
45
Surgery.
C. Ferrier Walters, t.K.C.b.
49
Ophthalmology.
A. Ogilvy, M.D., B.Ch., 13.A. JDud.
53
Reviews ot books
56
Editorial Notes
74
Notes on Preparations tor the oick
83
The Library of the Bristol Medico-Chirurgical Society
85
Meetings of Societies
Obituary Notices ...
93
Local Medical Notes
94

				

